# The envirome and the connectome: exploring the structural noise in the human brain associated with socioeconomic deprivation

**DOI:** 10.3389/fnhum.2013.00722

**Published:** 2013-11-12

**Authors:** Rajeev Krishnadas, Jongrae Kim, John McLean, G. David Batty, Jennifer S. McLean, Keith Millar, Chris J. Packard, Jonathan Cavanagh

**Affiliations:** ^1^Sackler Institute of Psychobiological Research, Institute of Health and Wellbeing, University of Glasgow, Gartnavel Royal HospitalGlasgow, UK; ^2^Department of Biomedical Engineering, School of Engineering, University of GlasgowGlasgow, UK; ^3^Medical Research Council Social and Public Health Sciences UnitGlasgow, UK; ^4^Clinical Epidemiology Group, Department of Epidemiology and Public Health, University College LondonLondon, UK; ^5^Glasgow Centre for Population HealthGlasgow, UK; ^6^Glasgow Clinical Research FacilityGlasgow, UK

**Keywords:** socioeconomic status, neighborhood deprivation, gray nodes, modularity, graph theory, cortical thickness

## Abstract

Complex cognitive functions are widely recognized to be the result of a number of brain regions working together as large-scale networks. Recently, complex network analysis has been used to characterize various structural properties of the large-scale network organization of the brain. For example, the human brain has been found to have a modular architecture i.e., regions within the network form communities (modules) with more connections between regions within the community compared to regions outside it. The aim of this study was to examine the modular and overlapping modular architecture of the brain networks using complex network analysis. We also examined the association between neighborhood level deprivation and brain network structure—modularity and gray nodes. We compared network structure derived from anatomical MRI scans of 42 middle-aged neurologically healthy men from the least (LD) and the most deprived (MD) neighborhoods of Glasgow with their corresponding random networks. Cortical morphological covariance networks were constructed from the cortical thickness derived from the MRI scans of the brain. For a given modularity threshold, networks derived from the MD group showed similar number of modules compared to their corresponding random networks, while networks derived from the LD group had more modules compared to their corresponding random networks. The MD group also had fewer gray nodes—a measure of overlapping modular structure. These results suggest that apparent structural difference in brain networks may be driven by differences in cortical thicknesses between groups. This demonstrates a structural organization that is consistent with a system that is less robust and less efficient in information processing. These findings provide some evidence of the relationship between socioeconomic deprivation and brain network topology.

## Introduction

Overlapping large-scale networks that are organized across the cortex form the anatomical and functional foundations of complex cognitive processes (Bressler and Menon, 2010). Complex network analysis based on graph theory has been recently used on neuroimaging data (MRI, MEG, and EEG) to explore different properties of these large-scale cortical network organization (Sporns, 2011). These studies have shown that human brain networks are optimally functioning systems that demonstrate small world properties, and a modular architecture (He et al., 2007; Bassett et al., 2008; Chen et al., 2008; Bullmore and Sporns, 2012). Modularity is an index of community structure within a large-scale network (Newman, 2006). That is, these networks have a tendency to form modules or communities with more connections between nodes within the module than between modules. Structurally, modules represent discrete entities whose functions are separable from those of other modules (Hartwell et al., 1999).

**Figure 1:**
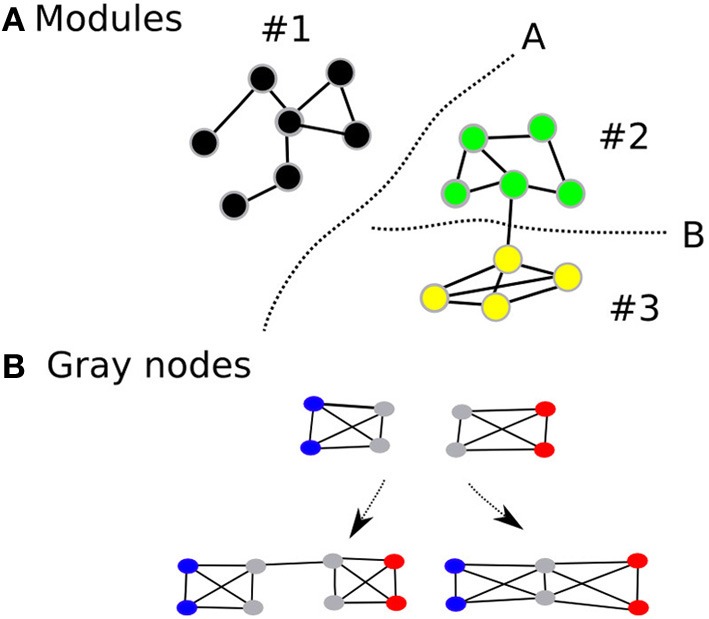
**Shows the modular architecture (A) and gray nodes (B)**. Gray nodes: Consider two fully connected networks **(B)**, with four nodes each and are fully connected. The two networks can be connected in two different ways. If they are connected as the first left in the bottom, then one additional edge is used. On the other hand, if they share the two nodes depicted in gray, then the combined module saves resources, i.e., there are two nodes and two edges less than the first combination. In addition, the average path lengths are shortened than the one with the non-sharing combination.

While modularity is usually associated with robustness of the network in biological systems, complex cognitive processes (an index of performance of the network) are unlikely to occur optimally within isolated modules (Hintze and Adami, 2008). Rather, they are likely to be dependent on the coordinated activity between several modules within the large-scale network. Indeed, most biological networks that survive in nature are those that achieve some balance between robustness and performance. Intuitively, it would be beneficial if the human brain network demonstrated modularity—increasing its robustness—but also had an architecture that facilitates efficient information transfer between modules—thereby improving performance. Therefore, while maintaining the advantages of having a modular architecture, we propose that the human brain will also demonstrate an overlapping modular architecture, where certain nodes (we call gray nodes) are included in many modules at the same time (Figure 1) (Zhao et al., 2011). Within an information processing system, such architecture, will improve information transfer between modules thereby increasing efficiency and performance of the network in terms of having lesser number of edges and shorter average path lengths. In short, while modularity represents the community architecture within a network, gray nodes represents an index of overlapping communities.

Survival in adverse environments may be associated with changes in network structure that make them less robust and reduce their performance. Neighborhood level socioeconomic status (SES) is associated with adversity and the presence of risk factors for reduced physical and neurocognitive health (Diez Roux and Mair, 2010; Srireddy et al., 2012). If indeed, cognitive functions are dependent on optimal functioning (and hence structure and topology) of large-scale brain networks, it is possible that SES is associated with changes in large-scale network structure. A small number of neuroimaging studies have shown SES to be associated with variations in individual brain anatomy and functional connectivity in adults (Gianaros et al., 2007, 2008). While network structure and topology have been found to be disrupted in a number of mental illnesses, no study has examined the relationship between neighborhood socioeconomic deprivation and brain network structure in humans.

The aim of the present study was to apply complex network analysis to examine the structural characteristics—modularity and gray nodes—of cortical networks derived from cortical morphology correlation (Figure 1). We also examined these structural characteristics in relation to socioeconomic deprivation. There is growing evidence that cortical morphology covariation is an indicator of connectivity between different regions of the brain (Worsley et al., 2005; Lerch et al., 2006; He et al., 2007; Bassett et al., 2008; Zalesky et al., 2010; Alexander-Bloch et al., 2013). Graph-theoretical network analyses based on morphological correlations have been used to examine brain network structure in healthy and clinical samples (He et al., 2007, 2009; Bassett et al., 2008).

Using complex network analysis of magnetic resonance imaging (MRI) surface-based morphometry we investigated the topological features of whole cortical anatomical networks in 42 neurologically healthy men from the most deprived (MD) and least deprived (LD) neighborhoods of Glasgow (Sporns, 2011). The connectivity matrices in the present study were derived from region-wise cortical thickness correlations between 68 anatomical parcellations and subjected to complex network analyses. We propose that the brain networks derived thus will show an overlapping modular architecture—by the presence of modules and gray nodes. We also examined to determine if these structural properties differed significantly between neurologically healthy people living in the most deprived (with higher risk of reduced mental health cognitive functioning) and the least deprived regions of Glasgow. Throughout the paper, “structural” refers to the network structure (e.g., modularity or proportion of gray nodes). We have used the term “anatomical” to refer to brain anatomy.

## Materials and methods

### Participants

Participants were recruited as part of a larger study (Psychological, social and biological determinants of ill health (pSoBid). Details of the design of pSoBid have been described elsewhere (Velupillai et al., 2008; Deans et al., 2009; Knox et al., 2012; McGuinness et al., 2012; McLean et al., 2012). Selection of participants was based on the Scottish Index of Multiple Deprivation 2004 (SIMD), which ranks small areas on the basis of multiple deprivation indicators across six domains, namely: income; employment; health; education, skills, and training; geographic access and telecommunications; and housing. Sampling was stratified to achieve an approximately equal distribution of the 666 participants across males and females and age groups (35–44, 45–54, and 55–64 years) within the most (bottom 5% of SIMD score) and LD areas (top 20% of SIMD score). Participants could opt-in for the neuroimaging component of the study. This paper presents the analysis from 42 male individuals who were randomly selected. This included 21 people from the most deprived regions and 21 from the least deprived regions, who were age matched.

### Image acquisition

All MR imaging were performed using GE Medical systems, 3T Signa Excite HD system (Milwaukee, USA) using an eight channel phased array (receive only) head coil. An axial 3D T1-weighted IR-FSPGR was acquired with TR = 6.8 ms; TE = 1.5 ms, Inversion Preparation time = 500 ms; Flip angle = 12°; FOV = 26 cm; Phase FOV = 70%; matrix: 320 × 320; 160 slices; Bandwidth 31.25 kHz; Slab thickness = 1 mm. The acquisition time for this scan was 8 min 54 s.

#### Cortical thickness measurements and parcellations

**Figure 2:**
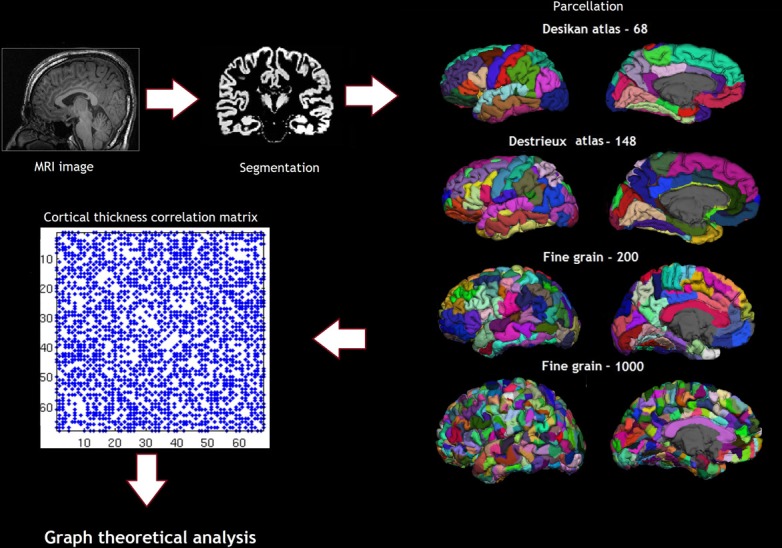
**Shows the pipeline of analysis, including the parcellation schemes—Desikan atlas and Destrieux atlas showing the sulcogyral parcellations and the Finegrain 200 and 1000 atlas as in Echtermeyer et al. (2011)**.

Cortical reconstruction was performed with the FreeSurfer image analysis suite, which is documented and freely available for download online (http://surfer.nmr.mgh.harvard.edu/). (Dale et al., 1999; Fischl et al., 1999; Fischl and Dale, 2000) Briefly, following skull-stripping and correction of inhomogeneity artifact, constrained region growing was used to create a unitary white matter volume for each hemisphere. The gray-matter/white-matter boundary for each cortical hemisphere was determined using tissue intensity and neighborhood constraints. The white matter surface was tessellated by assigning two triangles to the square face of each surface voxel. This process yielded approximately 160000 vertices per hemisphere. The white matter surfaces were deformed toward the gray matter/pial boundary, with a point to point correspondence at each vertex. Cortical thickness was computed as the distance between the white and the pial surfaces at each vertex. Cross-subject registration of hemispheric cortical surfaces was performed by projecting them onto the spherical representations. The maps produced are not restricted to the voxel resolution of the original images and are thus capable of detecting sub-millimeter differences between groups. The parcellations were obtained using the Desikan sulcogyral-based atlas, which follows the anatomical conventions of Duvernoy. The FS image-processing pipeline was visually inspected and corrected at critical points in order to avoid errors permeating through the subsequent analyses. Procedures for the measurement of cortical thickness have been validated against histological analysis and manual measurements. The Desikan Killiany atlas produces 68 parcellations based on gyri and sulci (Desikan et al., 2006). In addition to the Desikan Killiany atlas parcellation scheme, we also used fine-grained parcellation schemes based on anatomical sulcogyral boundaries including the Destrieux atlas, (148 parcellations) and fine-grained parcellation schemes (200, and 1000 parcellations) that did not follow anatomical conventions described in Echtermeyer et al. (Destrieux et al., 2010; Echtermeyer et al., 2011). The pipeline of the analysis and the parcellation are shown in Figure 2.

### Cortical thickness—between group comparison

Statistical comparisons of global data and surface maps were generated by computing a general linear model (GLM) of the effect of neighborhood deprivation (independent variable) on thickness (dependent variable) at each vertex in the cortical mantle, using the Query, Design, Estimate, Contrast (QDEC) interface of FreeSurfer. Age was used as nuisance covariate in the model. QDEC is a single-binary application included in the FreeSurfer distribution that is used to perform group averaging and inference on the cortical morphometric data produced by the FreeSurfer processing stream. (http://surfer.nmr.mgh.harvard.edu/fswiki/Qdec). Maps were created using statistical thresholds of *p* = 0.05 and were smoothed to a full width half maximum (FWHM) level of 20 mm. Since this analysis involved performing a GLM analysis at 160000 vertices, these maps were corrected for multiple comparisons by means of a cluster-wise procedure using the Monte Carlo Null-Z simulation method adapted for cortical surface analysis and incorporated into the QDEC processing stream. For these analyses, a total of 10,000 iterations of simulation were performed for each comparison, using a threshold of *p* = 0.05.

### Network construction

Network construction was based on parcellations of cortical thickness as described by He et al. (2007). We defined an anatomical connection (edge) as statistical associations in cortical thickness between cortical parcellations based on the Desikan Killiany atlas included in the FreeSurfer pipeline (nodes). The statistical similarity in cortical thickness between 2 regions was measured by computing the Pearson's correlation coefficient across subjects to create an interregional correlation matrix (*N* × *N*, where *N* is the number of brain regions based on Desikan cortical parcellation atlas, here *N* = 68). In order to keep the analysis as close as possible to previous reports, prior to the correlation analysis, a linear regression was performed at every region to remove the effects of age, and mean overall cortical thickness; the residuals of this regression were then substituted for the raw cortical thickness values (He et al., 2007; Chen et al., 2008). In order to be consistent with the cortical thickness group difference analysis presented above, the complex network analyses were repeated without mean overall cortical thickness in the model, but the results of our analysis did not differ significantly (results not shown). A separate matrix was produced for the MD (21 subjects) and the LD (21 subjects). As a first step, all negative correlations were discarded. As the correlation analysis was performed for all 68 × 68/2 = 1431 pairs of regions, we performed a multiple comparisons correction to test the significance of these correlations.

We applied the false discovery rate (FDR) procedure separately to each matrix in order to correct the multiple comparisons at a *q* value of 0.2 (this was chosen as at 0.05, both matrices were very sparse). (Genovese et al., 2002) Using this threshold, we constructed a symmetric connection matrix (Figures 5, 6), whose element was 1 if the cortical thickness correlation between 2 regions was statistically significant and 0 otherwise. This binarized connection matrix captures the underlying anatomical connection patterns of the human brain common to the population sample under study. We repeated all the analyses on matrices derived from the fine grained parcellation schemes described above, in order to validate our findings using multiple parcellation schemes.

### Modularity

All the modularity metrics were calculated on the above two adjacency matrices separately and compared to corresponding random networks. Modularity is an intuitional concept and there are variations in the mathematical definitions, where each has its own advantages and disadvantages. One common property among the various ways of defining modularity, however, is accounting for the agreed intuition about modularity, i.e., a module is a subset of nodes in a graph, whose connections among the elements within the subset are much denser than the ones to nodes outside the subset. Newman suggested the following modularity measure,*Q*:

$$Q=\underset{s\;\in \;S}{\max}\frac{1}{4m}s^{T}Bs,$$

where *s* is a column vector and element of the set *S*, *S* is the set of all column vectors whose dimension are equal to the number of nodes in the graph, *n*, and each component of the vector is either −1 or +1, (·)^*T*^ is the transpose. *B* is equal to *A* − *kk*^*T*^ /(2*m*), *A* is the adjacency matrix, whose dimension is *n* × *n*, and the *i*-th column (or row) and *j*-th row (or column) element is 1 (or 0) if *i*-th and *j*-th nodes are connected by an edge (or if there is no edge), *k* is a column vector whose element is the number of edges connected for each node, i.e., the degree of node, and *m* is the total number of edges. Roughly speaking, *B* quantifies the difference between the number of edges found in a subset of the given network structure, i.e., *A*, and the expected average from the random graphs, whose nodes degree is the same as the one of the given graph, i.e., *kk*^*T*^ /(2*m*). Hence, positive *Q* values imply that there are more edges found than the expected and it is, therefore, a module.

By obtaining *s* that maximizes the modularity, *Q*, the nodes are divided into two groups, i.e., modules, depending on the corresponding values in the maximizing vector, *s*. The maximization problem, however, is the integer quadratic programming problem, which is NP-hard. It is even computationally very difficult to obtain the true solution, which gives the global maximum value of *Q*. Note that *Q* is always less than or equal to 1. If the condition for *s* is relaxed so that it can take any real numbers, then the problem becomes finding maximum eigenvalue and the corresponding eigenvector of the matrix, *B*. This can be solved efficiently using the power-iteration, i.e., choosing an arbitrary initial vector, *s*_0_, and recursively updating the vector using *s*_*k+1*_ = *Bs*_*k*_ until it converges. Then, *s* maximizing *Q* is calculated simply by taking the sign of converged *s*_*k*_. To increase the chance of finding the global solution, these procedures are repeated a number of times with a different random initial vector, *s*_0_. If the calculated maximum value, *Q*, is positive (or negative), then the graph is divided (or declared indivisible).

Once the graph is divided into two modules, then each module is inspected whether it can be further divided by solving the following the maximization problem:

$$\Delta Q=\underset{r\;\in \;S^{g}}{\max}\frac{1}{4m}r^{T}B^{g}r,$$

where *r* is an element of the set *s*^*g*^, *s*^*g*^ is the set of *n*_*g*_-dimension column vectors whose element is either +1 or −1, *n*_*g*_ is the number of nodes in the module, which is found in the previous step, *B*^*g*^ is equal to *B*^*ij*^ − diag[*k*^*g*^], *B*^*ij*^ is a matrix constructed by a part of *B*, where the rows and columns belong to the module, *k*^*g*^ is the degree of each nodes only concerning *B*^*g*^, and diag [·] is the diagonal matrix, where the diagonal terms are given by the vector in the argument and the other elements are zero. Again, if Δ*Q* >0(or Δ*Q* ≤ 0), then the module is divided into two smaller modules (or declared indivisible). The above procedures are repeated on every module recursively until all modules are declared indivisible. By definition, the divisibility of a module is determined based on whether the modularity measure is positive or not. Very often, it is, hard to justify whether some subgroups of a graph are modules if the modularity contribution, i.e., *Q* or Δ*Q*, is very close to zero. As the mathematically possible maximum value is 1, the modular structure is much clearer if the modularity is closer to 1. Hence, the number of modules is calculated for various *Q*-threshold, which decides when modules are declared as indivisible.

### Gray nodes

A network, in general, is not a simple collection of modules but a combination of complicated overlapped modular structures, i.e., it demonstrates a hierarchical modular architecture. The overlapped modular structures are hard to decipher into elementary modules that pertain to the whole network. There are several methods to unravel the overlapping modular structure. In order to use a consistent measure with the modular calculation, an extended modularity (*Q*_*e*_) is defined as follows:

$$Q_{e}=\underset{s_{e}\;\in \;S_{e}}{\max}\frac{1}{4m}s_{e}^{T}Bs_{e},$$

where *s*_*e*_ is an element of the set, *S*_*e*_, and the set *S*_*e*_ is the collection of vector, *s*_*e*_, whose dimension is again, *n*, i.e., the number of nodes, and its element is either -1, +1, or 0. Compare to the vector *s* in *S*, *s*_*e*_ has one more degree of freedom in possible values (Zhao et al., 2011). The nodes corresponding to zero are called gray nodes, which are included in multiple modules at the same time or are not included in any module. Δ*Q*_*e*_ is defined in the similar manner. Gray node is a similar concept to that of connector hub and hierarchical or overlapping modular structure. While connector hubs are defined as nodes with greater than average degree of the network and distributed between both local and long range connections, gray nodes are defined as nodes that are shared by modules. It is an index of overlapping modular architecture of the network. Previous literature has described such overlapping architecture based on a prior definition of modularity by Newman and Girvan (Newman and Girvan, 2004; Nicosia et al., 2009; Lazar et al., 2010; Wang et al., 2012). On the other hand, “gray nodes” are a unified way to define the structure in the more recent modularity definition by Newman (Newman, 2006). This provides an advantage that we measure modular architecture, and the overlapping architecture using a consistent measure without requiring significant changes in the algorithm (Newman, 2006).

All calculations presented in this paper are based on Monte-Carlo simulations performed 1000 times. The distributions of all calculations are confirmed to be similar to Gaussian distributions (data not shown). Hence, there is no danger that the analyses based on the mean and the variance may give any false interpretations of the true distribution of the data. All graphs were compared to random graphs (with the same number of nodes and degree distribution as the corresponding brain networks).

## Results

Demographic details, differences in risk factors and performance on cognitive tests of the participants are shown in Table 1. In general, participants in the MD group had higher inflammatory and metabolic risk markers, poorer GHQ scores and performed poorly on a number of cognitive tests. Supplemental file shows the details of how early life and current individual level SES were derived. Table A1 shows that individual level SES covaried significantly with the neighborhood level deprivation status, and hence were not included in our data analysis.

**Table 1:** **Demographic and clinical characteristics of study participants**.

	**Least deprived *n* = 21 mean (s.d.)**	**Most Deprived *n* = 21 mean (s.d.)**	***t***	***p***
Age (years)	51.18 (8.7)	50.70 (8.75)	0.224	0.82
Alcohol units per week	15.81 (9.39)	18.61 (21.32)	−0.55	0.58
Diet score	95.24 (48.55)	40.66 (32.92)	4.26	< 0.001
GHQ 28 score	1.48 (2.71)	5.00 (5.59)	−2.59	0.015
NART errors	5.33 (3.719)	12.43 (6.66)	−4.26	< 0.001
Choice reaction time	860.14 (115.66)	1064.48 (168.6)	−4.5	< 0.001
Trail making test A	28.55 (7.59)	35.86 (12.97)	−2.18	0.035
Trail making test B	61.74 (20.81)	90.42 (29.98)	−3.4	0.002
RAVLT – trial 5	12.05 (1.74)	11.52 (2.06)	0.88	0.52
Cortisol (nmol/l)	354.37 (103.29)	398.63 (124.06)	−1.19	0.24
CRP (mg/L)	1.17 (1.34)	3.40 (2.94)	−3.16	0.004
ICAM (ng/ml)	234.48 (25.72)	309.67 (84.19)	−3.81	0.001
IL6 (pg/ml)	2.6235 (5.42)	2.5320 (1.76)	0.07	0.94
Fibrogen (g/L)	2.94 (0.61)	3.17 (0.95)	−0.89	0.37
D-dimer	89.81 (47.35)	150.32 (104.27)	−2.32	0.029
Glucose (mmol/L)	5.42 (0.57)	5.31(1.15)	0.38	0.70
HDL (mmol/l)	1.22 (0.20)	1.26 (0.36)	−0.46	0.64
Triglycerides (mmol/l)	1.71 (0.72)	2.29 (2.23)	−1.14	0.26
Insulin (uIU/ml)	7.1820 (4.82)	9.857 (6 8.43)	−1.23	0.22
Systolic BP (mmHg)	139.90 (17.03)	142.47 (20.96)	−0.43	0.66
Diastolic BP (mmHg)	81.28 (8.53)	82.85 (11.33)	−0.50	0.62
BMI (kg/m^2^)	27.02 (2.69)	28.42 (5.86)	−0.99	0.33
Waist-Hip ratio	0.90 (0.05)	0.97 (0.072)	−3.6	0.001
Intracranial volume (cc)	1572.94 (143.52)	1542.66 (161.72)	0.642	0.525

T, unpaired t-test; BMI, body mass index; CRP, C-reactive protein; IL-6, interleukin-6; ICAM-1, intercellular adhesion molecule.

### Cortical thickness differences between groups

**Figure 3:**
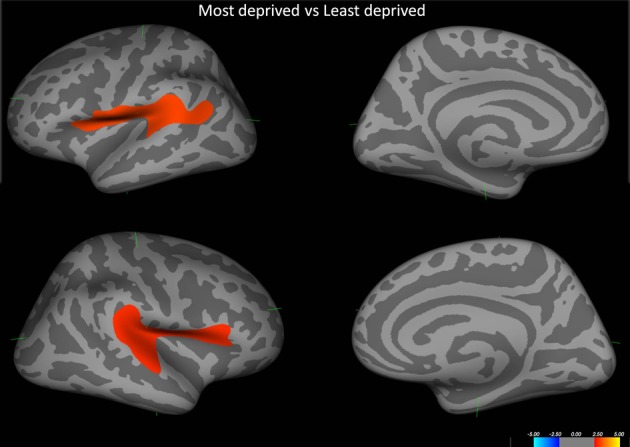
**Shows the difference in cortical thickness between the most deprived and the least deprived groups**. Red regions pertain to regions where the most deprived group showed cortical thinning. Covariates in the model—Age and alcohol use.

Initial analysis of cortical thickness across groups showed that those from the most deprived population had significant cortical thinning pertaining to bilateral perisylvian cortices. (Figure 3).

### Network analysis

**Figure 4:**
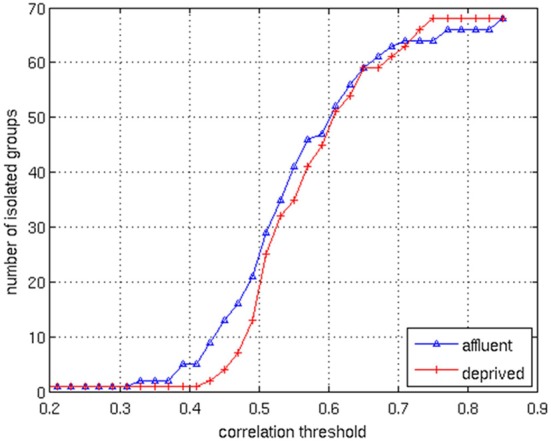
**The correlation values in the matrices are distributed between 0.1 and 0.9**. By changing the correlation threshold from 0.2 to 0.85, the number of isolated groups are counted for the both groups. The least deprived has more isolated groups than the deprived over the almost all values of the correlation threshold. Affluent: Least deprived; Deprived: Most deprived.

**Figure 5:**
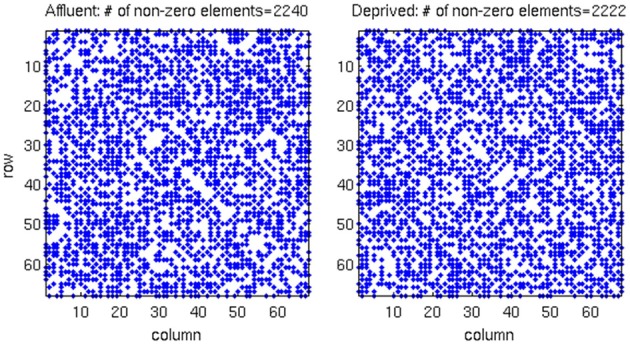
**The raw correlation matrix for each group shows that two groups have almost equal number of non-zero components in the matrix**. The correlation matrix for each group is given by a 68 × 68 matrix, where each value in the matrix is calculated from the cortical thickness correlation measured in 21 individuals. Affluent: Least deprived; Deprived: Most deprived.

**Figure 6:**
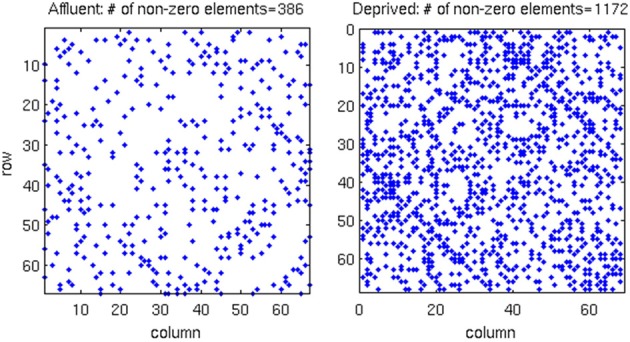
**In the correlation matrix for each group, all values below the FDR threshold are set to zero, where**. About three-times more edges survived the FDR procedure in the most deprived than the least deprived group. Affluent: Least deprived; Deprived: Most deprived.

**Figure 7:**
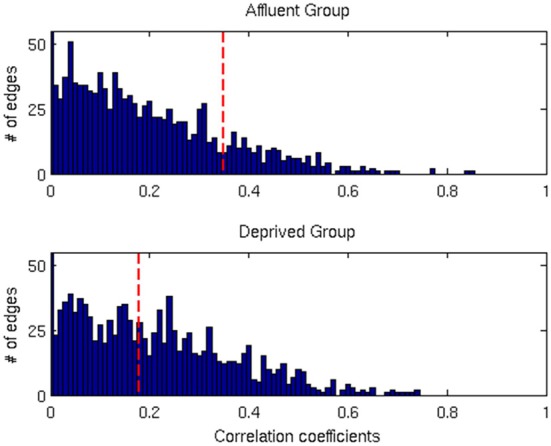
**The distributions of correlation coefficients for both groups**. The vertical red lines are the FDR threshold values for each group. Affluent: Least deprived; Deprived: Most deprived.

**Figure 8:**
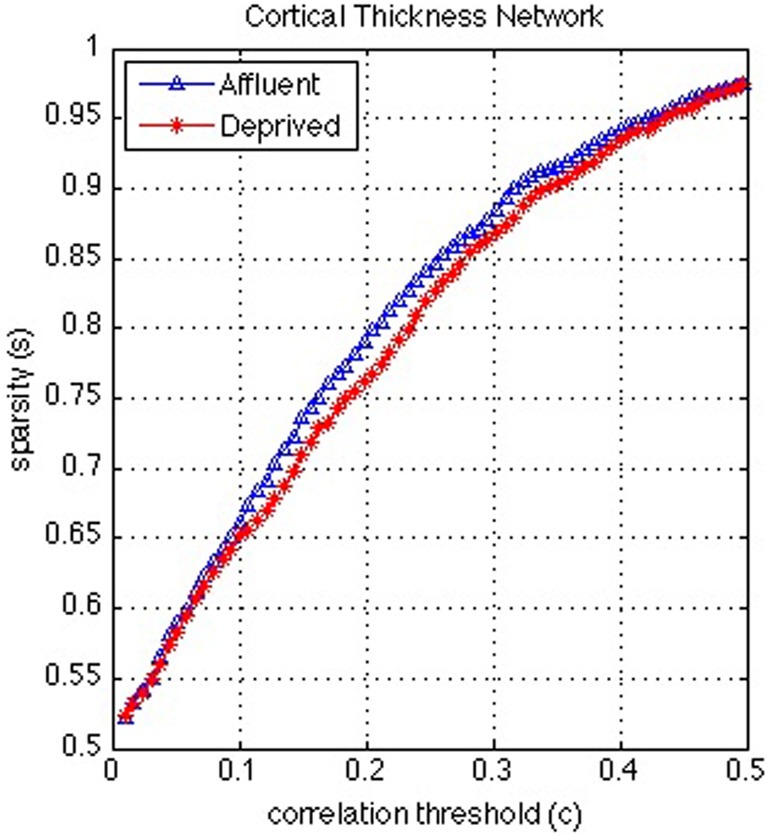
**Correlation and sparsity (Number of zeros divided by Maximum possible number of edges) relations in cortical thickness network**. The most deprived have more edges (denser network) than the least deprived for a fixed correlation threshold. On the other hand the least deprived would have more false positive edges than the deprived and/or the deprived would have more false negative edges than the least deprived for a fixed sparsity. Affluent: Least deprived; Deprived: Most deprived.

**Figure 9:**
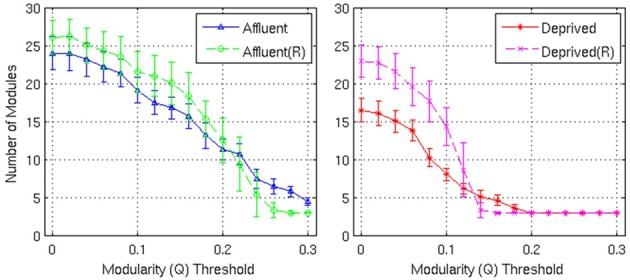
**Number of modules and the corresponding random graphs [indicated by “(R)”] with respect to various modularity (*Q*) threshold**. Error bars represent the 1σ-bound for each case. In the module calculation algorithm, if the module contribution, *Q* or Δ*Q*, is less than the threshold, it was declared indivisible. Higher thresholds imply strong modules. Affluent: Least deprived; Deprived: Most deprived.

**Figure 10:**
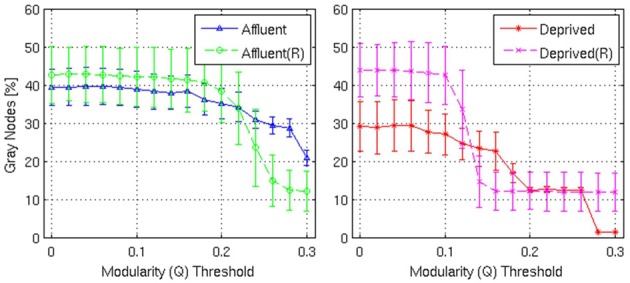
**Shows the proportion of gray nodes with respect to the corresponding Modularity threshold**. Error bars represent the 1σ-bound for each case. In the module calculation algorithm, if the module contribution, *Q* or Δ*Q*, is less than the threshold, it was declared indivisible. Higher thresholds imply strong modules. Gray nodes have two implications in the network structure: (i) efficient usage of resources and (ii) shorter average distance between nodes. Recycling existing nodes and edges to combine multiple modules saves limited resources to construct the network. It is believed that reducing wiring resources is one of the major selection pressure on the brain network evolution. Affluent: Least deprived; Deprived: Most deprived.

We conducted all analyses on binarised matrices derived from interregional correlations of cortical thickness. Initial examination of number of isolated modules showed that for a given correlation threshold, the least deprived group had greater number of isolated groups compared to the deprived group (Figure 4). The raw networks and FDR filtered networks are shown in Figures 5, 6. The distribution of the groups' correlation coefficients is shown in Figure 7. A direct comparison of the networks derived from the above populations, was not possible, as for a given correlation threshold, the sparsity (density) of the two networks were significantly different (Figure 8). In addition, the FDR procedure thresholded the two networks significantly differently. This method of thresholding resulted in different number of edges—k—(sparsity) in the networks of the two groups because of differences in their inter-regional cortical thickness correlations. We therefore compared the network structure derived from the groups to their corresponding random networks. The results of this analysis are shown in Figures 9, 10.

#### Modularity and grey nodes

Firstly, the networks derived from both groups showed a modular architecture, and the presence of gray nodes. Toward a modularity of 0.3 (strong modularity), the least deprived network had more modules, compared to its corresponding random network. However, the most deprived network, showed no difference from its random counterpart.

**Figure 11:**
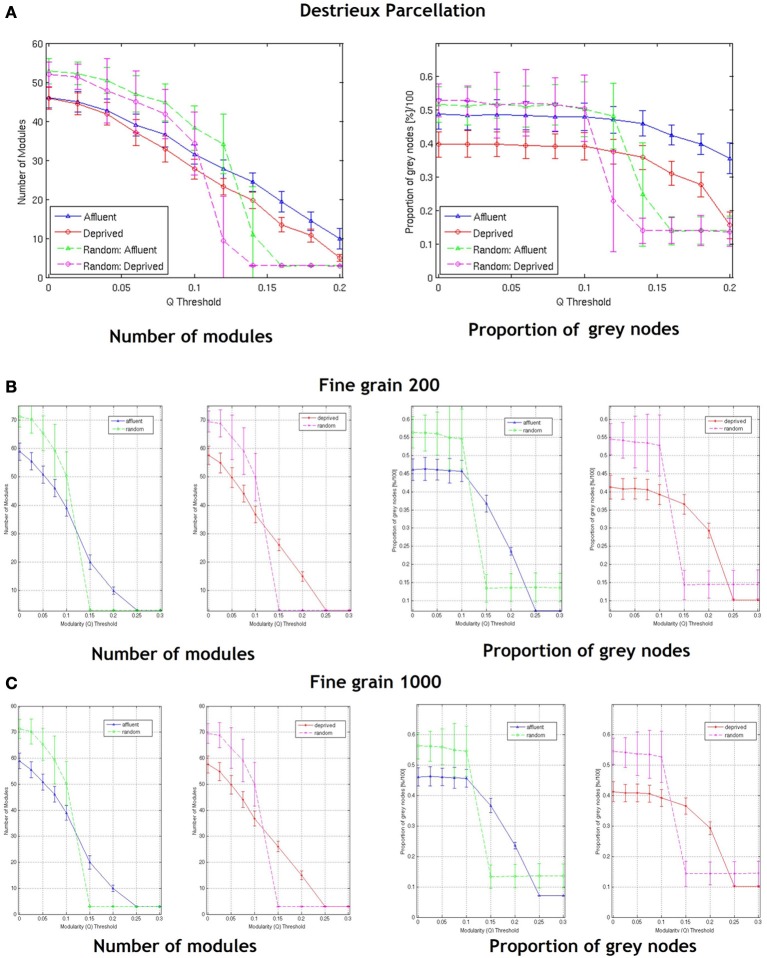
**Shows the number of modules and proportion of gray nodes at a fine grain level—(A)** parcellation following sulcogyral boundaries—Destrieux atlas (148 parcels) and **(B)** a parcellation scheme that does not follow the sulcogyral boundaries [**(B)** 200 parcels and **(C)** 1000 parcels). Affluent: Least deprived; Deprived: Most deprived.

With regards the gray nodes, for a given a modularity toward 0.3, the least deprived network showed significantly greater number of gray nodes compared to the corresponding random network. However, the most deprived network showed significantly smaller proportion of gray nodes compared to its random counterpart. While the differences between groups were maintained in the Destreaux atlas (148 parcels) that followed the sulcogyral boundaries, these differences were not seen with the finer grain parcellations of 200 and 1000 parcels that did not follow the sulcogyral scheme. (Figures 11A–C).

## Discussion

We have shown here that brain networks derived from cortical morphological correlations show a modular organization, and indeed an overlapping modular architecture as demonstrated by the presence of gray nodes. We have also shown that neurologically healthy subjects from the MD regions of Glasgow differ significantly in their brain network structure from those from the LD regions in comparison to their corresponding random networks on relatively coarse parcellations schemes that followed the sulcogyral boundaries. Brain networks in the MD group showed same number of modules and smaller proportion of gray nodes compared to their corresponding random network. These differences, however, disappeared at fine-grained parcellation schemes that did not follow the sulcogyral schemes.

A number of recent studies have shown that human brain network structure derived from anatomical covariance demonstrates a modular architecture (Chen et al., 2008, 2011). There are a number of advantages in having a modular architecture. Kaiser et al. suggest that this feature allows for low wiring costs; are time scale separable; allows for the coexistence of integration and segregation within a network; transient chimera states of resynchronization and synchronization; and also allows for rapid and robust assembly (Kaiser, 2007). In addition, a modular architecture is robust against random attacks on the network and helps to contain the effects of these attacks to the module, rather than spreading through the network.

We compared the brain network graphs with random graphs that had similar degree to the corresponding brain network. For both the LD and MD groups, at lower modularity thresholds, the brain network graphs had fewer modules compared to their corresponding random graphs. However, this phenomenon was reversed at higher thresholds. This is possibly because within the constraints of fixed resources (nodes/edges), brain networks enhance a few specific modules by rewiring and sacrificing unwanted modules.

In our study, for a given number of modules, the brain networks in the LD group showed stronger modular organization than their corresponding random graphs. In other words, the networks derived from the most deprived group had more edges between modules, which weakened the modular architecture. Previous work by Chen et al. using a similar technique showed that modules derived using correlations of cortical thickness, broadly gave out six functionally relevant modules (Chen et al., 2008). Using the same number (six modules) as Chen et al., the modules were functionally more relevant in the LD population (data not shown). For example, all anatomical regions pertaining to language function were integrated together within a given module. However, this was not the case with the MD. Anatomical regions pertaining to similar function were distributed across several modules, consistent with poor functional modular organization at a given threshold. While these modularity differences may be due to anatomical differences between groups that we have shown, these may have functional implications, as anatomical networks have been found to overlap with functional networks (Alexander-Bloch et al., 2013). If we consider these networks as information processing systems, then such a difference in network structure could contribute to greater noise and less efficient information processing within the system. However, a direct interpolation of the results of our study is not possible due to the static nature of our data.

We describe a new metric—gray node—as a measure of overlapping modular organization. While modularity improves the robustness within a system, it is unlikely that our brain network achieves optimal performance by operating as a number of different isolated modules. As stated previously, cognitive processes are likely to be the result of a number of modules interacting with each other in a fast and efficient way. The overlapping modular architecture—represented here by the presence of gray nodes—is beneficial in that given a fixed number of resources it provides the best modular architecture, maximizing the communication between modules thereby achieving a balance between robustness and optimal performance. Gray nodes have two implications in the network structure: i) efficient usage of resources and ii) shorter average distance between nodes. Recycling existing nodes and edges to combine multiple modules saves limited resources to construct an efficient network. It is believed that reducing wiring resources is one of the major selection pressures on the brain network evolution. Our results suggest that the networks derived from the MD group show much lower efficiency compared to their corresponding random network (Achard and Bullmore, 2007; Bullmore and Sporns, 2009). While metrics describing overlapping modules have been outlined previously, gray nodes have the advantage that it was derived from Newman (2006) and integrates well with the given modularity metric (Newman, 2006).

While the structural differences may be driven by the difference in cortical thickness between the two groups, the reason for the anatomical difference between the two groups is not clear. It should be noted that the groups differed on a number of variables that could potentially explain the observed difference. For example, those from the most deprived had poorer mental health and also had higher levels of inflammation. (See Table 1) We have previously shown inflammatory markers to be associated with cortical thickness (Krishnadas et al., 2013). We were, however, underpowered to explore the role of potential mediators that could explain the difference between groups in structural properties. Previous studies have demonstrated age related changes to modularity (Chen et al., 2011). Our groups were matched for age. Similarly, mental illnesses have shown to be associated with disruption to the modular architecture. A few studies have also examined this property in medical conditions like MS and epilepsy (He et al., 2009; Vaessen et al., 2012). A number of studies have shown an association between socioeconomic deprivation and brain anatomy and function in both children and adults, though none have examined the association with network structure (Gianaros et al., 2011; Hanson et al., 2011; Jednorog et al., 2012). A key question that remains is how these anatomical differences could contribute to poorer cognitive functioning and mental health. Interestingly, the MD group performed poorly on all cognitive tests, including NART (National adult reading test)—a test that is relatively stable through age, and often considered a test of measure of the peak achieved intellectual functioning. We did not examine if less modularity was directly associated with poorer cognitive functioning as utilizing correlation coefficients to construct the matrix meant that indices of modularity could not be calculated at an individual level. However, change in network structure is a potential mechanism by which regional anatomical brain deficits may contribute to global network topology, thereby resulting in poorer cognitive function. Previous studies have examined the relationship between intelligence quotient (IQ) and network properties. For example Li et al. found a significant positive correlation between number of edges and IQ. They also found that those with greater IQ had shorter path lengths, greater clustering coefficient (similar to our findings) and in general greater global efficiency of structural networks in the brain (Li et al., 2009). Similarly using resting state fMRI to examine the overall organization of the brain network using graph analysis, van den Heuvel et al. showed a strong negative association between characteristic path length of the resting-state brain network and IQ (Van Den Heuvel et al., 2009). They suggest that human intellectual performance is likely to be related to how efficiently the brain integrates information between various brain regions.

### Neighborhood level vs. individual level SES.

Socio-economic status (SES) refers to a multidimensional construct that is usually measured using a number of economic (e.g., income) and non-economic (e.g., education) indicators (Hackman et al., 2010). SES can be measured at an individual/household or at a neighborhood level. Regardless of the level of measurement (individual/neighborhood), SES has been associated with significant health disparities (Diez Roux and Mair, 2010). Most of the studies previously mentioned have examined the association between individual level SES and brain morphology. But individual level explanations for poor health do not capture significant social and structural determinants of ill health (Diez Roux and Mair, 2010). It is well-established that social circumstances have direct biological consequences, as well as impact on health behaviors (see Diez Roux and Mair for a detailed review on neighborhood deprivation). However, relatively small number of studies have explored the contributions of individual level SES indicators with neighborhood level indicators to health inequalities. Neighborhood level deprivation has been associated with poor health outcomes due to inequalities in resource distribution. These neighborhoods have physical (e.g., access to food) and social (e.g., violence) attributes that are contributors to health outcomes. However, individual and neighborhood deprivation are likely to interact significantly. For example, Stafford and Marmot found that living in a deprived neighborhood has the most adverse impacts on poorer individuals possibly because they are more dependent on collective resources of the neighborhood (Stafford and Marmot, 2003). In our study, individual level SES covaried significantly with neighborhood level SES. (For details of this analysis see Table A1 in Appendix) Due to the nature of the sampling technique, people from the most deprived neighborhoods also had poorer individual SES. This is partly because neighborhood deprivation scores (SIMD) are derived from data pertaining to individuals in the area. Since our groups differed inherently in their individual SES, it was deemed inappropriate to co-vary for the effects of individual SES (Miller and Chapman, 2001). Our relatively small sample size was also not sufficiently powered to examine if individual SES contributed significant variance over and above that explained by neighborhood SES or vice versa. The extreme group sampling technique prevented us from examining any dose-response effect of either individual or neighborhood level deprivation in our sample.

### Effect of parcellation scheme on network structure

Zalesky et al. have previously shown that network topology vary considerably as a function of the spatial scale of the atlas used (Zalesky et al., 2010). Previous reports that have examined cortical thickness covariance network structure in clinical and non-clinical populations have used the same parcellation scheme (Desikan-Killiany atlas) used in our study (Raj et al., 2010; Hanggi et al., 2011; Romero-Garcia et al., 2012; Yang et al., 2012). Of note, Romero-Garcia et al. in order to examine the effect of network resolution on topological properties, compared the Desikan-Killiany atlas based parcellation with finer parcellation schemes of up to 1494 parcellations (Romero-Garcia et al., 2012). Interestingly they found that highly grained cortical scales showed enhanced local connectivity (clustering coefficient), and local efficiency, but increased path length and decreased global efficiency. Our findings resonate that of Romero-Garcia et al., in that, at different parcellation schemes, the network topologies differed (Romero-Garcia et al., 2012). For fine-grained parcellation schemes that did not follow sulcogyral boundaries, the LD brain network, and MD brain network were similar. At a modularity threshold of around 0.3, both network structural properties looked similar to their random counterparts (suggesting a decrease in global properties at more fine grained schemes) (Figures 1A,B).

Anatomically, since cortical thickness is a continuous measure, regions that lie close to each other will show very similar cortical thickness and hence high correlation. Here, a fine parcellation schemes, may uncover local connection (or a forking-U fiber connection), while a coarse may not (see Figure 1 in Zalesky et al) (Zalesky et al., 2010). In addition, regions close to each other are likely to be anatomically connected by the tangential neurons and dendrites. It is possible that in our case, the group differences disappeared when geometrically close connections were exposed at the finer parcellation schemes. In addition, at finer parcellation, where the number of parcels far exceed the number of subjects in the study, the study may have been significantly underpowered to show significant differences between groups (Zalesky et al., 2010).

It is also possible that network structure derived from relatively coarse parcellations are more representative of large scale cortical networks, while the networks derived from the fine-grained parcellations also include the meso/microscale connections representing regional/local connections. Whatever the case, it is clear that the granularity of chosen parcellations may affect the results of the network analysis. Our data suggest that when exploring connectivity, choosing the right granularity that is best suited to answer the question of interest is vital. However, clear cut guidelines pertaining to this are absent. One suggestion is that in order to answer clinical questions, anatomically relevant atlases like AAL or the sulcogyral parcellations (FreeSurfer) as used in our study may be more relevant. Interestingly for a finer (than Desikan atlas) parcellation that follows the sulcogyral boundaries (the Destreaux atlas—149 parcellations), the difference between the brain and random networks in the most deprived group disappear at around a modularity threshold of around 0.2 (Figure 11A).

### Sparsity (density) and modularity

Although we found significant differences between the networks and their corresponding random graphs, we did not perform a direct comparison of the network structure between the two groups, as the thresholds imposed by the FDR correction led to matrices that were significantly different in their sparsity (density). Thresholding a matrix is a problem when comparing networks that have different sparsity for a given correlation coefficient (Van Wijk et al., 2010). While the reason for the sparsity difference between the groups is not known, revealing topological differences gives deeper insights into the difference in networks than just revealing the sparsity difference. One recommended way to solve this problem is by fixing the sparsity (density) of a matrix, and comparing the networks at the same fixed sparsity threshold (Hanggi et al., 2011). This approach will, however, increase the false negative or false positive correlations at a given threshold. For instance, in our case, at more than 90% of correlation thresholds, the LD network was more sparse (less edges—k) than the MD. i.e., for a given correlation threshold, the networks from both the groups were different in their size (the number of edges). The difference in modularity between groups may therefore be k dependent. This difference in correlation threshold may have arisen from anatomical difference in the bilateral perisylvian cortical thickness we found between groups. While these morphological differences could have led to a reduction in correlation between regions that are actually connected, this could also have led to an increase in the number of spurious correlations (false positive), between regions that are not biologically connected, thereby contributing to noise within the network. Therefore, introducing false edges by fixing the sparsity was not thought to be meaningful.

### Cortical thickness correlation as a measure of connectivity

While the biological meaning of structural covariance is not clear, structural covariance networks have been found to be genetically heritable, associated with cognitive function, recapitulate functional networks, and change over the life span. See Alexander-Bloch et al. (2013) for a detailed recent review of this literature (Alexander-Bloch et al., 2013). Cortical volume is a construct that is derived from two distinct properties of the cortical sheet: cortical thickness and surface area and have distinct cellular and genetic basis. Rakic's (2007, 2009) radial unit hypothesis proposes that symmetrical cell division within the neural stem cell pool in the ventricular zone causes an exponential increase in the number of radial columns—that result in surface area (SA) expansion. This is independent of asymmetrical cell division in the founder cells that is responsible for a linear increase in the number of neurons within a radial column, contributing to cortical thickness (CT) (Rakic, 2007). Complex network analysis using graph theory using cortical structural covariance networks derived from CT and cortical SA shows different structural properties, suggesting that they contribute to different properties within cortical networks (Sanabria-Diaz et al., 2010). Cortical gray matter volume is almost entirely driven by differences in the cortical SA rather than CT. (Im et al., 2006) Secondly, recent large scale studies have shown that these two parameters—CT and SA—have independent genetic basis (Panizzon et al., 2009). Thirdly, life course trajectories of these cortical parameters seem to be different. While gyrification—a ratio of total SA to pial SA remains fairly stable post childhood through to early adulthood, CT changes dynamically through this period (Rathbone et al., 2011; Raznahan et al., 2011; Salinas et al., 2012). However, more recent studies suggest that the relation between age and cortical parameters in adulthood, are complex (Hogstrom et al., 2012). CT in addition appears to be highly susceptible to various environmental influences over the life course such as smoking, alcohol dependence, and marijuana use while SA appears to be influenced by various unique developmental factors (Kuhn et al., 2010; Lopez-Larson et al., 2011; Momenan et al., 2012). This highlights the importance of studying volume and thickness independently in morphometric studies (Winkler et al., 2010). Surface area appears to be influenced by various unique developmental factors and is less susceptible to age-related differences in later life (ref). These and other findings suggest that while cortical surface areas increase significantly prenatally and remain fairly stable post childhood, cortical thickness changes dynamically across the lifespan (Raznahan et al., 2011; Salinas et al., 2012; Shaw et al., 2012). We restricted our analysis to cortical thickness as we were examining the association between what an environmental variable (deprivation) and a cortical parameter (cortical thickness) that has previously shown to be influenced by environmental factors. Further analysis using other parameters may reveal differences in structural properties that are contributed by factors that may be influenced early in life.

### Limitations

While the positive features of this study include a well-characterized community based cohort, there are limitations to be acknowledged: the cross-sectional design limits our ability to attribute causation and there is some selection bias in that the participants opted in. We did not include any sub-cortical regions particularly those that are relevant to physiological stress response. Smaller sample size meant that there was a potential for type 2 error, especially with regards the fine grain parcellations. We excluded female subjects in order to reduce variance in cortical morphology pertaining to gender. Further work would involve replication of the study in a larger population, including younger population, targeting critical periods of brain growth. Finally, future work to develop a clearer biological framework of a more comprehensive investigation of metabolic and inflammatory markers may be more informative.

In summary, people from the MD population show less modular and overlapping modular architecture of the brain networks derived from cortical morphology compared to their corresponding random graphs at a coarse sulcogyral parcellation scheme. At fine grained parcellation scheme that did not follow sulcogyral boundaries, this difference disappeared. While the difference in network structure at the coarse level may be the result of anatomical differences at a large scale level, the exact etiopathogenesis and the consequence of this difference is not clear. Taken together we propose that brain networks associated with MD group may be less efficient in information and signal processing at a large scale level. Future studies should look at longitudinal functional and effective connectivity studies using MRI and EEG/MEG to explore the effect of socioeconomic status on development.

## Author contributions

Rajeev Krishnadas and Jongrae Kim are joint first authors who contributed equally to this work. Rajeev Krishnadas and Jongrae Kim analyzed data and wrote the paper. John McLean created the MRI protocol and analyzed the data. All other researchers were involved in designing, performing the research and discussing the paper.

### Conflict of interest statement

The authors declare that the research was conducted in the absence of any commercial or financial relationships that could be construed as a potential conflict of interest.

## Appendix

### Early life and current socioeconomic status (SES)

Correspondence analysis was used to explore the factor structure of early and late SES. This is similar to factor analysis for categorical data. These analyses confirmed that markers of early and late SES are well-represented by single factors, and determined the corresponding weight associated with each level of each marker. By taking levels with positive and negative weights as representing relative deprivation or affluence, the following cut-offs were used to derive early and late SES scores: Early life SES (ESES) consisted of the following items: number of siblings (> 3 = 0), people per room (> 1 = 0), paternal social class (IIIM or below = 0), parental housing tenure (Not owner = 0), use of a car by the family (no car = 0). The current SES (CSES) score was derived from current income (< 25k = 0); current social class (III or lower = 0); current housing tenure (not owner = 0). For each variable, those deemed to be least deprived scored 1 and those deemed to be most deprived scored 0. These scores were then summed for each, giving total score (0–5 for ESES, 0–3 for CSES), higher scores suggesting more affluence. The components are shown in the Table A1.

**Table A1:** **Individual level SES**.

		**Affluent (*N* = 21)**	**Deprived (*N* = 21)**	**^*^*p***
Childhood overcrowding (no. people per room at age 11)	*n* (missing)	21 (0)	21 (0)	
	≤1	12 (57.1%)	3 (14.3%)	0.0088
	>1	9 (42.9%)	18 (85.7%)	
Fathers social class	*n* (missing)	21 (0)	19 (2)	
	I	5 (23.8%)	1 (5.3%)	0.0042
	II	7 (33.3%)	0 (0.0%)	
	IIIM	4 (19.0%)	11 (57.9%)	
	IIINM	3 (14.3%)	3 (15.8%)	
	IV	2 (9.5%)	2 (10.5%)	
	V	0 (0.0%)	2 (10.5%)	
Parents tenure status at age 11	*n* (missing)	21 (0)	21 (0)	
	Owner	12 (57.1%)	0 (0.0%)	<0.001
	Not owner	9 (42.9%)	21 (100.0%)	
Parents owned car at age 11	n (missing)	21 (0)	21 (0)	
	Yes	12 (57.1%)	4 (19.0%)	0.0247
	No	9 (42.9%)	17 (81.0%)	
Number of siblings	*n* (missing)	21 (0)	21 (0)	
	0–2	16 (76.2%)	13 (61.9%)	0.5055
	3 or more	5 (23.8%)	8 (38.1%)	
Current social class	*n* (missing)	21 (0)	20 (1)	
	I	11 (52.4%)	0 (0.0%)	<0.001
	II	8 (38.1%)	3 (15.0%)	
	IIIM	0 (0.0%)	8 (40.0%)	
	IIINM	2 (9.5%)	2 (10.0%)	
	IV	0 (0.0%)	6 (30.0%)	
	V	0 (0.0%)	1 (5.0%)	
Current income	*n* (missing)	20 (1)	21 (0)	
	<£15,000	0 (0.0%)	8 (38.1%)	<0.001
	£15–25,000	1 (5.0%)	9 (42.9%)	
	£26–35,000	1 (5.0%)	2 (9.5%)	
	£36–45,000	3 (15.0%)	2 (9.5%)	
	> £45,000	15 (75.0%)	0 (0.0%)	
Current tenure status	*n* (missing)	21 (0)	21 (0)	
	Owner Occupier	20 (95.2%)	5 (23.8%)	<0.001
	Tenant	1 (4.8%)	16 (76.2%)	

* *Fishers exact test*.
